# Supplementary material for: Examining holistic processing strategies in dogs and humans through gaze behavior
Source: PLoS One. 2025 Feb 19;20(2):e0317455. doi: 10.1371/journal.pone.0317455 (PMC11838905; doi:10.1371/journal.pone.0317455)
Supplement: Attachment — Submitted filename: Response to Reviewers.pdf [file pone.0317455.s001.pdf]

Dear Reviewer #1,

Thank you for your thorough review and kind feedback on the manuscript. Especially, we appreciate you pointing out the critical error in Figure 3. We have addressed the issues you raised as follows:

1. Figure Placement: The images in the "else upper half" and "else lower half" columns of Figure 3 have been corrected and are now properly positioned.
2. Figure Resolution: All figures have been replaced with higher-resolution versions for improved clarity.

Thank you again for taking the time to review our manuscript.

On behalf of all authors,
Sincerely,
Soon Young Park
